# A study on image quality provided by a kilovoltage cone‐beam computed tomography

**DOI:** 10.1120/jacmp.v14i1.3888

**Published:** 2013-01-07

**Authors:** Julia Garayoa, Pablo Castro

**Affiliations:** ^1^ Servicio de Radiofísica Hospital Universitario Puerta de Hierro – Majadahonda Majadahonda Madrid Spain

**Keywords:** image quality, cone‐beam CT, on‐board imager (OBI), IGRT, ART

## Abstract

The image‐guided radiotherapy technique (IGRT) makes use of imaging devices to verify the positions of the target volume and organs at risk during the treatment sessions. In this work we evaluate the image quality provided by an imaging system based on a kilovoltage cone‐beam CT, and explore its ability to perform IGRT and adaptive radiotherapy. We analyze the accuracy of the image slice width, the spatial resolution using the MTF function, the image uniformity, the signal‐to‐noise ratio, the contrast‐to‐noise ratio, the low‐contrast sensitivity, and the HU linearity with density. The studied parameters are evaluated in an objective and quantitative way, allowing for a direct comparison with other imaging devices. We conclude that the analyzed cone‐beam imaging system is adequate to accurately perform IGRT within its clinical use, despite the high level of noise present in a cone beam caused by scatter. We also point out the presence of a bowtie wobble artifact in the reconstructed images. Nevertheless, we conclude that these features do not limit the capability of the system to perform adaptive radiotherapy in most cases.

PACS number: 87.57.‐s

## I. INTRODUCTION

Successful external radiotherapy requires the ability to reproduce in the treatment room the patient setup used in the simulation. The employment of imaging devices to verify the patient position is known as image‐guided radiotherapy technique (IGRT). Therefore, an image of the patient at the time of treatment is acquired and registered with the reference image obtained in the simulation. Once the best match between both sets of images is achieved and the proper setup of the patient is checked, it is possible to correct the patient position by shifting the treatment table.

Thus, IGRT imaging systems are designed to detect and correct positioning errors in every treatment session. The conventional method consists in the acquisition of two orthogonal planar megavoltage images (2D) with an electronic portal imaging device (EPID).^(^ ^1^ ^)^ The comparison between these images and the digitally reconstructed radiographs (DRR) leads to an anatomic registration based on high‐contrast areas such as bony structures. However, this method does not discriminate soft tissue. Some modern imaging systems, such as those based on a kilovoltage cone beam (kV CBCT) allow obtaining tomographic images of the patient that can be directly compared with the CT planning study. Moreover, they also provide information of low‐contrast structures, which make possible the registration based on areas with soft tissue.

Another possibility is to use these tomographic images in the monitoring of patients which may present significant changes in the treatment volume or anatomical changes that could modify their outer contour. Such modifications of the initial conditions would in turn alter the dose distribution both within the treatment volume itself and the organs at risk. kV CBCT images may also be used to perform adaptive radiotherapy (ART), a computation of the treatment dose distribution, and/or contouring structures and treatment replanning.^(^ ^2^ ^,^ ^3^ ^,^ ^4^ ^,^ ^5^ ^,^ ^6^ ^)^ Reliable contouring of organs at risk and treatment volumes requires an appropriate image quality, as well as accurate and reproducible HU if dose calculations are going to be performed.^(^ ^7^ ^,^ ^8^ ^,^ ^9^ ^)^

Clearly, imaging systems play an increasingly relevant role in radiotherapy. As a consequence, it becomes necessary to understand their characteristics and limitations, as well as to implement a quality assurance program that includes these imaging systems.^(^ ^1^ ^,^ ^10^ ^,^ ^11^ ^)^ In general, the tests to be performed can be classified in the following groups: functional and safety, geometric, and image quality. It is also important to know the calibration procedures, which directly affect the behavior of the system and aim to reduce possible artifacts or improve the quality of the acquired images.

The system analyzed in this study is the Varian on‐board imager (OBI) in the mode cone‐beam computed tomography (CBCT). The manufacturer provides a set of calibration procedures that include the system geometric calibration and image detector calibration itself. Geometric calibration aims to correct errors associated with the non‐rigid nature of the system (such as source‐detector misalignment or nonisocentric gantry rotation). The imaging system detector calibration includes several tasks: creation of a defective pixel correction map, acquisition of a dark field, measurement of the detector response to a uniform radiation field, measurement of the charge trapping effect, creation of normalization maps over a homogeneous phantom to consider scattered radiation and beam hardening effect, and HU calibration. These calibration procedures define corrections that are directly applied to the acquired images and contribute to improve the quality of the reconstructed images. For instance, the presence of defective pixels can lead to the appearance of ring artifacts, which can be minimized with a proper calibration of the imaging system. The calibration should be performed for each acquisition protocol under the following circumstances: when changes that may affect the imaging system occur, and when a decrease in image quality is observed which, according to manufacturer's instructions, should be checked monthly. Although the manufacturer provides a calibration for all acquisition modes, the user can also perform them, if necessary.

Safety and functional tests, as well as geometric tests, have been already described in the literature. Yoo et al.^(^ ^10^ ^)^ determined the OBI's mechanical precision to be 1.5 mm for the isocenter localization and less than 1 mm for the arms positioning accuracy, with time stability below 1 mm in both cases on a follow‐up period of 8 months. In relation to image quality, low‐ and high‐contrast resolutions have been studied in a qualitative way.^(^ ^9^ ^,^ ^10^ ^,^ ^12^ ^)^

In order to avoid subjectivity in image quality tests, it is possible to determine physical parameters that characterize the system objectively — namely, the modulation transfer function for spatial resolution, and the contrast detail ratio, calculated based on statistical criteria, for low‐contrast resolution.

In this paper, we present a study of the volumetric CBCT image quality obtained with the OBI system to test its capability to perform both IGRT and ART. Among other parameters, system spatial resolution and low‐contrast resolution are analyzed. A comparison with the results obtained with a multislice CT scanner dedicated to simulation is also included.

## II. MATERIALS AND METHODS

Image quality tests have been performed on the tomographic CBCT images provided by the OBI (software Version 1.5). The OBI system (Varian Medical Systems Inc., Palo Alto, CA) consists of an X‐ray tube and an amorphous silicon flat‐panel detector, both mounted on the robotic arms (Exact, Varian Medical Systems Inc.) and orthogonally coupled to the MV beam of the CLINAC 21EX (Varian Medical Systems Inc.) linear accelerator. It provides an X‐ray cone beam that allows obtaining volumetric images using a filtered back‐projection reconstruction algorithm.

The flat panel, composed by an amorphous silicon detector coupled to a CsI:Tb scintillator, has a 397 mm×298 mm active area, and consists of a 2048×1536 detector matrix with a distance between detectors of 194 μm. The system has a 10:1 ratio focused antiscatter grid to reduce the amount of scattered radiation reaching the detector.

The OBI system has two working modes: full‐fan and half‐fan. In the full‐fan mode the detector is centered on the axis of rotation and is used to visualize regions with a small diameter or small soft tissue organs, such as the prostate. In the half‐fan mode, the detector is shifted in a direction perpendicular to the kilovoltage X‐ray beam, increasing the scanned area, hence enlarging the diameter of the field of view from 25 cm, as in the full‐fan mode, to 45 cm. In both cases, a filter must be placed at the exit of the X‐ray tube. The full‐fan filter (the one used in the full‐fan mode) is symmetric with respect to the beam axis in the axial plane, being narrower at the center. The half‐fan filter is asymmetric, narrow in the central part with increasing thickness towards the edge of the X‐ray beam. These filters absorb part of the incident radiation, and they are used to compensate for the difference in the thickness traversed by the beam on the patient, so that the photon fluence that reaches the detector is as uniform as possible. In addition, the absorption of low energy X‐rays reduces the dose received by the patient and the noise generated in the detector. An image artifact known as bowtie wobble, related to the movement of these filters with gantry rotation, has been reported in the literature.^(^ ^13^ ^)^

The CBCT images are acquired with the Pelvis clinical protocol. This protocol acquires 650 projections in a 364° gantry rotation with the detector placed at 150 cm from the source and acquisition technique of 125 kVp, 80 mA, 13 ms pulse width (1 projection per pulse) and half‐fan filter. The focal spot size used is 0.8 mm×1.1 mm. The radiation field as measured at the isocenter is 27.2 cm×20.6 cm, and the reconstruction matrix is 512 × 512. The field of view chosen for this study is 256 mm × 256 mm, smaller than the one used in the clinical practice, so that the Nyquist frequency (1 line pair per mm) lies beyond the cut‐off frequency (maximum 1 pl/mm for all the analyzed protocols and reconstruction filters). The reconstructed image slice width is set to 2 mm with a Ram‐Lak convolution filter and medium ring artifact suppression filter. The standard reconstruction filter is employed, although results obtained using other filters (sharp and smooth) are also discussed. Finally, the results obtained for the Pelvis Spot Light protocol with full‐fan filter, used for prostate treatment in clinical practice, are briefly discussed. In this case, gantry rotates 200° and acquires 375 projections with a pulse width of 25 ms.

Regarding the simulation CT Aquilion LB (Toshiba Medical Systems, Otawara, Japan), it is a multislice CT scanner with a detector matrix consisting of 40 detector rows and 16 acquisition channels. The detectors located in the 16 central rows are 0.5 mm wide as measured at isocenter, while the other 24 rows, 12 on each side, are 1 mm wide. The detectors are separated by septa that absorb part of the scattered radiation reaching the detector. The analyzed images were obtained using the routine clinical protocol for pelvis, adapting some parameters to match the ones used by the OBI in order to make a comparison between both systems in conditions as similar as possible. Therefore we used the following protocol: helical technique with 120 kV, pitch 0.938, a detector configuration of 2 mm × 16 acquisition channels, gantry rotation period of 1 s, and focal spot size of 0.9 mm×0.8 mm. The reconstructed images are 2 mm wide, with a 512 × 512 pixel matrix and a field of view of 256 mm (the usual slice width is 3 mm and the FOV is around 450 mm). In clinical practice, a modulated tube current is used, with a tuning based on the scanograms acquired prior to helical scan. In the present study, a fixed tube current set to 80 mA is used to match the one used by OBI in Pelvis protocol. It is worthwhile to comment that this value is similar on average to the one obtained in real patients.

Therefore, the technique described above is employed to obtain the CT images used for contouring and treatment planning with IMRT in pelvic regions. They are also used as reference for comparison with CBCT images acquired in the treatment unit. The IGRT protocol followed in our hospital for this kind of treatment consists in performing a CBCT in all treatment sessions prior to irradiation. Thus, it is an online protocol, which implies daily correction of systematic and random errors. Additionally, patient preparation is verified (for instance, full bladder and empty rectum for prostate treatment).

Apart from the geometric differences between both systems (source‐to‐detector distance, 150 cm in CBCT versus 127.5 cm in CT), one of their main differences is the fan beam size along the z‐axis. The maximum fan‐beam size available in the CBCT is 20.6 cm as measured at isocenter, while the largest fan‐beam width for the CT is 3.2 cm. As a consequence, the amount of scattered radiation is much higher in the CBCT. Both systems incorporate an antiscatter grid and correction algorithms to minimize its effect. The CBCT larger coverage in the z‐axis allows obtaining the data in a single X‐ray tube rotation, being the acquisition time close to 1 min. In the case of the CT scanner, it is necessary to use several rotations around the patient over the moving table in order to cover the same range. However the acquisition time is smaller in this case (around 15 s) because of the faster tube rotation. Z‐axis resolution is limited by the fan‐beam width, determined by the detector configuration used. For this reason, the influence on the z‐axis resolution of the fan‐beam width, from 2 mm × 16 channels (3.2 cm fan‐beam width) to 0.5 × 16 (0.8 cm fan‐beam width) has been analyzed.

Our study is based on images acquired with the Catphan 600 phantom (The Phantom Laboratory Incorporated, Salem, NY). The phantom, cylindrical in shape, is constructed of PMMA and consists of 5 modules designed to perform various quality tests in tomographic images.^(^ ^14^ ^)^ The phantom long axis (z‐axis) has to be placed longitudinal to the CT table, the modules are in transverse planes to the phantom z‐axis (x–y plane).

**Figure 1:**
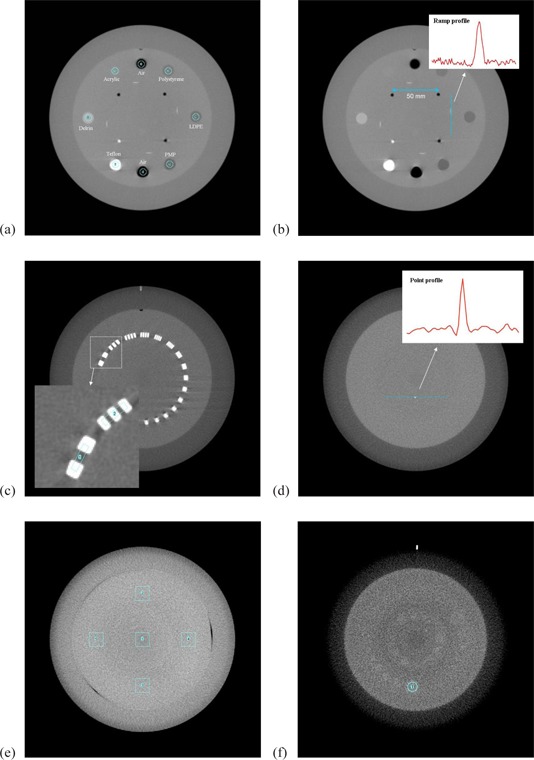
Images of the Catphan 600 phantom acquired with the CBCT system: (a) CTP404 module: HU verification, (b) CTP404 module: spatial linearity and pixel size verification, and slice width measurement, (c) CTP528 module: modulation transfer function (MTF) determination using the bar pattern, (d) CTP591 module: modulation transfer function determination using the point spread function, (e) CTP486 module: HU uniformity, (f) CTP515 module: low‐contrast sensitivity evaluation.

Module CTP404 has a set of inserts made of materials with different densities whose HU are in the range [−1000, 990], so the linearity of the system (HU density) can be checked (Fig. 1(a)). The spatial linearity is verified in this section measuring the known distance between four small inserts made of Teflon and air (Fig. 1(b)). This module also contains four wires rotated 23° with respect to the phantom x–y plane, which are used to measure the image slice width (Fig. 1(b)). Module CTP528 contains a set of bar patterns with different spatial frequencies (Fig. 1(c)). Module CTP591 has a tungsten‐carbide bead embedded into a uniform material, used to evaluate spatial resolution (Fig. 1(d)). Module CTP486 has uniform density equivalent to water (Fig. 1(e)). Finally, module CTP515 consists of groups of inserts with different size and nominal contrast: 1%, 0.5%, and 0.3%, that can be used to evaluate low contrast sensitivity (Fig. 1(f)).

Digital images, stored in DICOM format, are automatically analyzed by a set of ImageJ^(^ ^15^ ^,^ ^16^ ^)^ macros specifically designed for this purpose. In the following we describe the performed image quality tests.

The automatic analysis of the images requires a correct phantom positioning as well as an appropriate image quality, as recommended in the Catphan user manual.^(^ ^14^ ^)^ The correct phantom positioning is checked by defining a threshold pixel value to localize some reference points in the CTP404 module which are used to measure the phantom rotation angle around its long axis. Images rotated more than 1° are rejected, as described below. This criterion is relevant to estimate the uncertainty of our measurements. The regions of interest (ROI) used to perform the tests are generated automatically; therefore, a phantom rotation could cause an inappropriate ROI localization.

### A. Geometric distortion and pixel size verification

The small air and tungsten inserts in CTP404, which separated a known distance of 50 mm (see Fig. 1(b), are automatically localized. Then the geometric distortion both in the x‐ and y‐axis is evaluated using the measured distance between the inserts. The two main factors influencing this parameter are the divergence of the X‐ray beam and the magnification factor.

### B. Slice width

The accuracy of the selected slice width is evaluated with the ramp method using the four ramps (2 verticals and 2 horizontals) placed inside the CTP404 module. These ramps are rotated 23° around the x‐y plane. The slice width is given by FWHM·tan (23°), where FWHM is the full width at half maximum of the ramp profile. We report the average slice width value measured over the four ramps.

### C. Z‐axis spatial resolution: SSP

Z‐axis spatial resolution is measured using the slice sensitivity profile (SSP). A profile of the tungsten insert from the CTP591 module is performed over a sagittal reconstruction plane. The FWHM of the generated profile is registered. In addition, the impact of the fan‐beam size on the z‐axis resolution has been analyzed, from 2 mm × 16 channels (3.2 cm fan‐beam width) to 0.5 × 16 (0.8 cm fan‐beam width).

### D. X‐Y plane spatial resolution: MTF

The modulation transfer function (MTF) measures the system spatial resolution. The MTF will be obtained in two ways: from the image of a point object and from a bar pattern with different spatial frequencies. A spatially invariant linear system is assumed in both cases, implying that there is no overlap of system response in frequency space. This hypothesis has been experimentally verified both for the CBCT and the CT, measuring the system response to a point impulse at different image planes and at different locations within the same image plane. For each point we have studied the two directional components, radial (line between isocenter and point) and azimuthal (tangential to the radial direction), which may be affected by various design parameters.^(^ ^17^ ^)^

#### D.1 Point object

The MTF of a spatially invariant linear system is the modulus of the object transfer function (OTF):

(1) MTF(u,v)=|OTF(u,v)|

where

(2) $$OTF(u,v)=\frac{H(u,v)}{H(0,0)}$$

and *u, v* are the frequency space coordinates. The function *H*(*u,v*) is the system transfer function, defined as the Fourier transform of the point spread function (PSF), which is the system response to a point‐like object (δ‐Dirac function):

(3) H(u,v)=TF(PSF(x,y))

Therefore, it is possible to extract the MTF from the system response to a point‐like object (δ‐Dirac).^(^ ^18^ ^)^ The CTP591 module contains a tungsten bead, which acts as a point object.

The determination of the MTF function from the system response to a point object (PSF) will be carried out in two ways.

##### D.1.1 2D Fourier transform of PSF image (PSF 2D)

Once the image of the point object (PSF) has been obtained, fluctuations present in the background nearby the point are removed. Then, the 2D Fourier transform of the resulting PSF is performed. Pixel values in radial and azimuthal directions are obtained from the generated frequency spectrum corresponding to ${\text{MTF}}_{\text{rad}}$ and ${\text{MTF}}_{\text{azim}}$, respectively.

##### D.1.2 1D analytic fit of the PSF (PSF 1D)

The image system response to a point‐like object is a Gaussian function with width σ, the Fourier transform of which is another Gaussian with width 1/σ.^(^ ^18^ ^)^ We analyze both point profiles (radial and azimuthal directions), fit the obtained data to a Gaussian function and register the $\sigma_{\text{rad},\text{azim}}$ widths. Hence, the MTF is given by the Gaussian function centered at the origin with a width of 1/σ.

To account for the influence of the bead finite size in the MTF measurement with both methods, the following correction is applied:^(^ ^19^ ^)^

(4) $$F(u)=2\frac{J_{1}(\pi ud)}{\pi ud}$$

where $J_{I}(x)$ is the first order Bessel function, *u* is the frequency, and *d* is the tungsten sphere diameter. The correction factors for each spatial frequency are shown in Table 1.

**Table 1:** Correction factor for the bead finite size applied to calculate the modulation transfer function using the point spread function. We show the value of the correction factor corresponding to various spatial frequencies.

$u(cm^{-1})$	*F(u)*
1	1.0010
2	1.0039
3	1.0086
4	1.0156
5	1.0246
6	1.0356
7	1.0489
8	1.0646
9	1.0826
10	1.1033

#### D.2 Bar pattern

The MTF can be understood as the image to object contrast ratio. In fact, it can be shown that MTF(u) represents the system modulation of a *u* frequency sinusoidal input. Therefore, if one has a sinusoidal pattern, the image modulation created by the system becomes a direct measurement of the MTF.

Phantoms providing a sinusoidal pattern are unusual, but phantoms with a square pattern of different spatial frequencies are quite common. According to Fourier's Theorem, a square wave can be expressed as a sum of sinusoidal waves; thus, the MTF can be calculated based on the system modulation of a square bar pattern.^(^ ^20^ ^)^ The MTF written in terms of the system response to square waves with different spatial frequencies is:^(^ ^21^ ^)^

(5) $$MTF(u)=\frac{\pi }{4A_{0}}\left(A(u)+\frac{A(3u)}{3}-\frac{A(5u)}{5}+\frac{A(7u)}{7}+\ldots \right)$$

where *u* is the line frequency, *A(u)* is the output amplitude of the *u* frequency square wave, and $A_{0}$ is the amplitude of the input square wave.

The relation between amplitude and variance for a sinusoidal wave is given by $M^{2}=A^{2}/2$. For a square wave, on the other hand, this relation becomes M=A. Therefore:

(6) $$MTF(f)=\frac{\pi \sqrt{2}}{4M_{0}}\left(M(f)+\frac{M(3f)}{3}-\frac{M(5f)}{5}+\frac{M(7f)}{7}+\ldots \right)$$

As a consequence, the MTF is measured as the standard deviation (square root of variance) of a set of ROIs centered in the different inserts of the bar pattern present in the CTP528 module.

As we already mentioned, in our case phantom positioning is important, as the ROIs are automatically generated assuming that the phantom is perfectly centered. We found that phantom rotations above 1° introduce an error in the MTF‐50% which is higher than 3%. This is why our program rejects images rotated more than 1°. To perform this test, we intentionally rotated the position of the ROIs used in the MTF calculation and compared the result with the one obtained with the correct ROI orientation.

### E. Image uniformity and signal‐to‐noise ratio

We evaluate image uniformity and the signal‐to‐noise ratio using the CTP486 module. Image uniformity has also been analyzed with the normalization phantoms provided by Varian to perform the CBCT image calibration. They are two cylindrical homogeneous phantoms, one of 25 cm in diameter made of polyethylene and the other of 45 cm in diameter made of polyurethane.

Image nonuniformity is analyzed in two ways. We study the profiles generated over the uniform section of the Catphan phantom and Varian normalization phantoms. In addition, we calculate the integral nonuniformity defined over 5 squared ROIs (side length of 15 mm) located in the center and the periphery of the image, in the four cardinal points (see Fig. 1(e):

(7) $$UI=m_{\max}-m_{\min}$$

where $m_{\max}$ and $m_{\min}$ are the maximum and minimum ROI pixel mean values, respectively. Signal‐to‐noise ratio is measured over the same ROIs, and it is defined as the ratio between the ROI mean pixel value $m_{i}$ and its standard deviation $\sigma_{i}$:

(8) $$SNR=\frac{\left|m_{i}\right|}{\sigma_{i}}$$

(i: center, north, south, east, west).

Finally, we evaluate another parameter that reveals the existence of cupping or capping effect,^(^ ^11^ ^)^ since it compares the pixel mean value in the image center and in the periphery:

(9) $$C=m_{c}-m_{p}$$

where $m_{p}$ is the average of the mean pixel values measured at the ROIs in the periphery. A negative C value indicates cupping, while a positive value indicates capping.

### F. Contrast‐to‐noise ratio and low‐contrast sensitivity

Contrast‐to‐noise ratio is measured in the largest insert with the highest nominal contrast (1%) provided by the low‐contrast test module CTP515 (see Fig. 1(f). The contrast‐to‐noise ratio is defined as:^(^ ^11^ ^,^ ^22^ ^)^

(10) $$CNR=\frac{\left|m_{i}-m_{b}\right|}{\sqrt{\sigma_{i}^{2}+\sigma_{b}^{2}}}$$

where *i* corresponds to the low contrast insert, *b* to the background measured in a region next to the insert, *m* denotes the mean value of the corresponding ROI, and σ its standard deviation.

Low‐contrast sensitivity is characterized using the contrast‐detail curve. According to Rose model,^(^ ^23^ ^,^ ^24^ ^)^ an object is distinguishable from its background if the object‐to‐background signal ratio exceeds some threshold. This threshold defines a contrast‐detail curve. Objects with size and contrast above the curve are distinguishable, while those below are indistinguishable from the surrounding background. In order to find the contrast‐detail curve, the contrast of the inserts present in the phantom must be evaluated, as well as the image noise measured in the phantom uniform section.^(^ ^17^ ^,^ ^25^ ^)^

The noise associated to an insert of certain size is measured as the standard deviation of the mean values of 28 ROIs of the same size defined in the Catphan uniform section. Since the phantom provides low‐contrast inserts of nine different sizes ranging from 2 to 15 mm in diameter, nine groups of 28 ROIs of the corresponding size are created. The contrast of each insert is obtained as the difference between the mean pixel values of the ROI centered in the insert and another ROI placed in the background next to the insert. The smaller the ROI, the higher the standard deviation, making it more complicated to distinguish an object with HU close to the background HU.

Some references establish a statistical criterion to objectively decide whether the objects of a given size and contrast are distinguishable from the background.^(^ ^17^ ^,^ ^25^ ^)^ In particular, they claim that a low‐contrast object with a mean pixel value which differs from its background mean pixel value in less than 3.29 standard deviations is undistinguishable with a 95% confidence level.

### G. HU density curve

The HU corresponding to different materials present in the CTP404 module are measured as the mean pixel value of a circular ROI with a 4 mm radius centered in those materials.

The materials of the different inserts are made of commercial plastics. There are also two air inserts. In Table 2 we show the relative density provided by the manufacturer for each material and the expected HU. These values correspond to the ones used in the system calibration.

**Table 2:** Materials present in the CTP404 module from the Catphan 600 phantom. The relative density of each material and its corresponding HU reference value, provided by the manufacturer, are shown.

*Material*	*Relative Density* ^*a*^	*Reference HU*
Air	0.00	−1000
PMP	0.83	−200
LDPE	0.92	−100
Water	1.00	0
Polystyrene	1.05	−35
Acrylic	1.18	120
Delrin	1.41	340
Teflon	2.16	990

a Water as reference.

## III. RESULTS

In this section we analyze the results obtained for the CBCT system and the CT scanner. The results presented correspond to five sets of images acquired the same day; this permits us to evaluate the reproducibility of the performed tests. We show the mean values and their corresponding standard deviations, unless otherwise stated. Furthermore, in order to analyze the temporal stability of the studied parameters, we have performed the proposed image quality assurance program weekly during three months.

### A. Geometric distortion and pixel size verification

Geometric distortion is not observed in the reconstructed tomographic images by any of the two systems. The mean value of the detected error in the measurement of known distances (50 mm) is 0.4±0.2mm for the CBCT and 0.1±0.1mm for the CT. In both cases, the error is smaller than the pixel size, 0.5 mm. No significant time variations were observed (variation range: 0.0, 0.5 mm).

### B. Slice width

The nominal slice width of the images is 2 mm, while the measured value using the ramp method over the CBCT images for the standard reconstruction filter is 2.2±0.1mm. For the CT, with a detector configuration of 2 × 16, the measured slice width is 2.8±0.1mm. Table 3 shows the measured slice width in the CBCT images for the three studied reconstruction filters, and in the CT images for various acquisition techniques. As seen from Table 3, if we consider a narrower X‐ray beam in the CT scanner so that the data are stored with a higher z‐axis spatial resolution (for instance 0.5×16 or 1 × 16), a better slice width accuracy is achieved. No time trend was observed (variation range: 2.1, 2.3 mm).

**Table 3:** Slice width and FWHM of the slice sensitivity profile. The nominal slice width of the images acquired is 2 mm. The results obtained for various reconstruction filters (CBCT) and detector configurations (CT) are shown.

*Acquisition Parameters*	*Slice Width (mm)*	*FWHM (mm)*
CBCT ‐ Sharp	1.9±0.1	2.7±0.1
CBCT ‐ Standard	2.2±0.1	2.8±0.1
CBCT ‐ Smooth	2.2±0.1	3.3±0.1
CT ‐ 0.5×16	2.0±0.1	2.3±0.1
CT ‐ 1×16	2.1±0.1	2.1±0.1
CT ‐ 2×16	2.8±0.1	3.7±0.1

### C. Z‐axis spatial resolution: SSP

SSP function is characterized through the FWHM of a longitudinal profile defined over the tungsten insert in a sagittal slice; the results are shown in Table 3. Z‐axis spatial resolution is considerably higher for the CBCT than for the CT, under the studied conditions. Again a narrower X‐ray beam is needed in the CT in order to achieve a similar spatial resolution in both systems.

According to the results shown in Table 3, the reconstruction filter employed in the CBCT system affects the z‐axis spatial resolution, being higher for the sharp filter.

Finally, we state that this parameter does not present significant changes over time (SSP variation range: 2.3, 3.1 mm).

### D. X‐Y plane spatial resolution: MTF

Both studied systems have been considered to be spatially invariant with a real input function, so the obtained MTF is symmetric. Hence we only present the results corresponding to the positive frequency axis.

**Figure 2:**
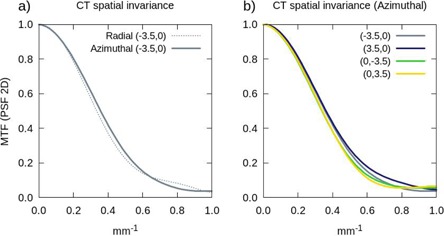
CT spatial invariance: (a) equivalency between the azimuthal (solid) and radial (dashed) components of the modulation transfer function (MTF) determined with the point spread function with the point object located at (−3.5, 0.0); (b) equivalency between the azimuthal components of the MTF determined with the point spread function for various locations of the point object: (−3.5, 0.0), (3.5, 0.0), (0, −3.5), and (0, 3.5).

The spatial invariance has been tested by comparing the system response to a point object (PSF 2D method) placed in several positions (x, y) of the FOV, namely: (0,±2), (0,±2.5), (± 2, 0), and (± 3.5, 0) cm. From Fig. 2, one can state that, in the CT system, the directional MTF components for the positions (0,±2.5) and (± 3.5, 0) are coincident within the experimental uncertainty, for each localization and between different positions within the same image plane. In other positions and for the OBI system, we obtained similar results; therefore, we do not show them.

In the following we will show the average MTF of both components, radial and azimuthal.

Coinciding with published results,^(^ ^11^ ^)^ no change in the MTF function was found when changing the image plane (z=± 3 cm).

**Figure 3:**
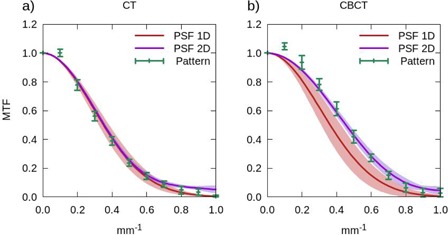
Modulation transfer functions (MTF) obtained with the three methods considered: 1D analytical fit of the point spread function (PSF 1D: red), 2D Fourier transform of the point spread function (PSF 2D: purple), and the bar pattern method^(^ ^20^ ^)^ (Pattern: green). Plot (a) shows the results obtained in the CT, and plot (b) the ones corresponding to the CBCT. The solid lines represent the mean values of the five consecutive measurements carried out, and the error bars (Pattern) or shaded regions (PSF) denote their first standard deviation.

The MTF obtained using the three different methods described in the previous section, for both the CBCT and the CT, are shown in Fig. 3, where the measured mean values and their corresponding standard deviations are displayed. The bar pattern method is inconsistent with the two PSF methods in the low frequency range, as the pattern only provides a valid approximation for frequencies over one‐third of the cutoff frequency.^(^ ^20^ ^)^ The reproducibility of the method based on the analytical fit of the PSF (PSF 1D) is poor because of the uncertainty of the method, mainly determined by the reduced number of points employed in the curve fitting. In addition, if the system does not show a Gaussian response to a point object, one should find an appropriate fitting function and analytically solve the Fourier transform integral, which is time consuming and implies an additional computational cost. For these reasons, and since the PSF 2D method presents a better reproducibility (lower standard deviation), it is taken as the reference method.

**Figure 4:**
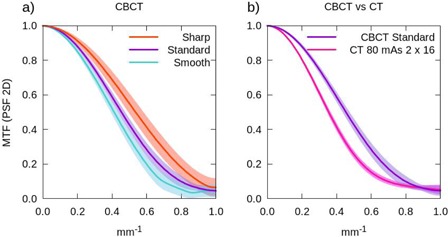
Modulation transfer function (MTF) (a) obtained with the PSF 2D method in the CBCT system for the three studied reconstruction filters: sharp (orange), standard (purple), and smooth (light blue); (b) comparison between the MTF functions corresponding to the CBCT with the standard reconstruction filter (purple) and the CT with 80 mAs and the detector configuration 2 × 16 (pink). The solid lines represent the mean values of the five consecutive measurements carried out, and the shaded regions denote their first standard deviation.

We consider that the agreement shown by the different methods is good for the CT (see Fig. 3(a) in the whole frequency range, except for the lower frequencies where, as mentioned above, the bar pattern method has a limitation. For the CBCT system (Fig. 3(b)), the MTF obtained with the analytical fit method (PSF 1D) presents a discrepancy at all frequencies, a behavior which is not observed in the CT. We guess that this difference may be due to the reduced number of points with which the curve fitting is performed, around 4 points in the OBI and 8 in the CT. Moreover, the influence on the spatial resolution of the reconstruction filter employed has been analyzed. Fig. 4(a) shows the MTF obtained with the smooth, standard and sharp filters with the PSF 2D method. The solid line represents the measured mean value and the shaded region corresponds to the 1 σ standard deviation. The smooth reconstruction filter presents the lowest spatial resolution in the whole frequency range explored, while it has the highest signal‐to‐noise ratio, as we will discuss below. On the contrary, images reconstructed with the sharp filter show higher spatial resolution at all frequencies.

Finally, in Fig. 4(b) we compare the MTF functions obtained with both systems, CBCT and CT. The CBCT spatial resolution is somewhat higher than that of the CT, under the conditions adopted in the present study. This can be related to the smaller size of the detectors present in the CBCT system.

No time trend was observed from the data.

### E. Image uniformity and signal‐to‐noise ratio

**Figure 5:**
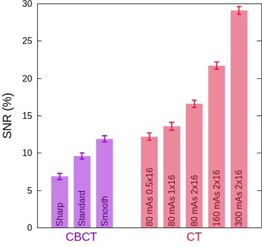
Signal‐to‐noise ratio (SNR) for the CBCT (purple) using three reconstruction filters (from left to right: sharp, standard, and smooth), and for the CT (red) using several combinations of tube current and detector configurations (from left to right: 80 mAs 0.5×16, 80 mAs 1 × 16, 80 mAs 2 × 16, 160 mAs 2 × 16, and 300 mAs 2×16).

The image uniformity and SNR of the CBCT images for any of the studied reconstruction filters is significantly lower than that obtained with the simulation CT scanner. This is due to the increased presence of noise, since a cone beam generates more scatter. In Fig. 5 we show the SNR values obtained with the ROI located in the image center of the uniform section of the phantom for both the CBCT, with the three studied reconstruction filters, and the CT, for different cone beam sizes and tube currents. In Table 4 we show the uniformity index (Eq. (7)) and the C parameter (Eq. (9)) which indicates the presence of cupping effect.

**Table 4:** Uniformity index and C parameter, measured in the uniform section CTP486 of the Catphan 600 phantom. The results obtained for various reconstruction filters (CBCT) and detector configurations and/or tube currents (CT) are shown.

*Acquisition Parameters*	*UI (HU)*	*C (HU)*
CBCT ‐ Sharp	21±3	−15±1
CBCT ‐ Standard	21±3	−16±1
CBCT ‐ Smooth	21±3	−15±1
CT ‐ 80 mAs 0.5×16	1.3±0.5	1.0±0.6
CT ‐ 80 mAs 1×16	1.8±0.5	1.1±0.6
CT ‐ 80 mAs 2×16	1.3±0.5	0.4±0.6
CT ‐ 160 mAs 2×16	2.0±0.5	0.4±0.6
CT ‐ 300 mAs 2×16	1.5±0.5	0.1±0.6

The CBCT images present cupping effect, meaning that the central part of the image is hypodense compared to the periphery. For instance, for the CBCT system with the standard reconstruction filter C=−16±1, while C=0.4±0.6 for the CT. The cupping effect is related to the beam hardening correction, which is partly corrected by the system software, and the significant presence of scatter (which can be corrected with an antiscatter grid). As a consequence, and also due to ring artifacts, the OBI images are less uniform than the CT images, as reveals the UI parameter. The presence of ring artifacts in the reconstructed images may be due to differences in the detector gains or to charge trapping effects. The OBI system incorporates a correction to mitigate this effect, as well as a processing filter that reduces these kinds of artifacts. We have used the medium ring artifact suppression filter, as recommended by the manufacturer.

In Fig. 5 the influence of the reconstruction filter on the CBCT images is shown. The use of the sharp filter increases the noise, and therefore the SNR decreases, while the smooth filter enhances the SNR by reducing the noise. For the CT, the impact of the fan beam size and the tube current has been studied. As can be seen in Fig. 5, the narrower the fan beam, the higher the noise contribution, decreasing the SNR. On the other hand, an increase in the tube current results in an increment in the SNR proportional to the square root of the tube current increment. The reconstruction filter or the acquisition technique does not affect significantly the incidence of the cupping effect or the measured image nonuniformity in any of the two systems.

**Figure 6:**
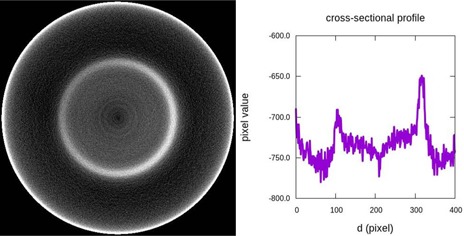
CBCT tomographic image for the Varian uniform phantom (left) and a crosssectional profile (right). The effect of bowtie wobble appears as a bright ring.

The uniformity of the CBCT images was also analyzed with the normalization phantoms provided by Varian, which have the advantage of being larger than the Catphan. We obtained similar results in both normalization phantoms, therefore we only discuss the results obtained with the larger one. In Fig. 6 we show the phantom image obtained with the OBI and the corresponding profile centered in the phantom. The images were obtained with the standard medium reconstruction filter. The profile shows, as we already mentioned, a signal decrease in the central part and various ring artifacts. A bright ring of radius around 22 cm is also present. This artifact appears when the half‐fan filter is used and does not depend on the phantom or its position. We suppose that this is the same type of bowtie wobble artifact described in the literature for the full‐fan filter.^(^ ^13^ ^)^ The artifact appears always in the same position, coinciding with the projection of the filter on the image detector. In the Catphan phantom, the artifact remains unnoticed because this phantom is smaller (radius of 20 cm) and the ring is beyond its outer edge. The image quality tests performed with the Catphan phantom are restricted to the center of the FOV. The obtained results could change in the periphery of the FOV due to the artifacts mentioned above.

Finally, we have studied the image uniformity obtained with the CBCT in the full‐fan mode with the Pelvis Spot Light protocol, which is the one used in the prostate IGRT clinical protocol in our hospital. There is a reduction in the SNR value from east to west due to the bowtie wobble artifact related to the movement of the filter with gantry rotation.^(^ ^13^ ^)^ This artifact is a ring with an increase of signal at one end of the ring and a decrease in the opposite end.

No significant time variations were observed.

### F. contrast‐to‐noise ratio and low‐contrast sensitivity

**Figure 7:**
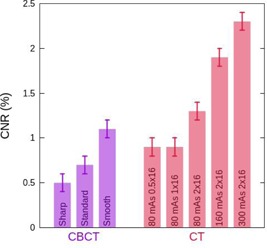
Contrast‐to‐noise ratio (CNR) for the CBCT (purple) using three reconstruction filters (from left to right: sharp, standard, and smooth) and for the CT (red) using several combinations of tube current and detector configurations (from left to right: 80 mAs 0.5×16, 80 mAs 1 × 16, 80 mAs 2 × 16, 160 mAs 2 × 16, and 300 mAs 2 × 16).

**Figure 8:**
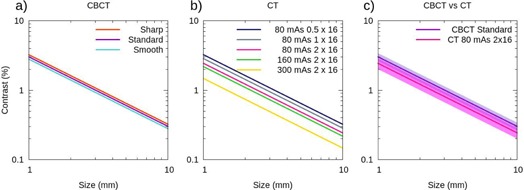
Contrast‐detail curve. Plot (a) shows the curves for the CBCT obtained with three reconstruction filters: sharp (orange), standard (purple), and smooth (light blue). Plot (b) shows the curves for the CT obtained using several combinations of tube current and detector configurations: 80 mAs 0.5×16 (dark blue), 80 mAs 1 × 16 (grey), 80 mAs 2 × 16 (pink), 160 mAs 2 × 16 (green), and 300 mAs 2 × 16 (yellow). Plot (c) shows a comparison between the contrast‐detail curves obtained in the CBCT with the standard reconstruction filter (purple) and in the CT with 80 mAs 2 × 16 (pink). The solid lines represent the mean values of the five consecutive measurements carried out, and the shaded regions denote their first standard deviation. The standard deviations are not displayed in plots (a) and (b) to make the plots clearer.

The CNR (Eq. (10)) has been measured in the largest insert with 1% nominal contrast available in the Catphan phantom, the contrast‐detail curve has been calculated, too. The results are shown in Figs. 7 and 8 for both the CBCT and the CT for the various reconstruction or acquisition techniques examined. In Fig. 8(a), we show the contrast‐detail curve corresponding to the CBCT with the three reconstruction filters, in Fig. 8(b) the ones corresponding to the CT for different acquisitions, and in Fig. 8(c) we compare both systems.

Let us now focus on the results obtained for the CBCT images with the standard filter and for the CT images acquired with 80 mAs and the detector configuration 2 × 16. The CBCT system exhibits a lower CNR value, CNR=0.7±0.1 for the CBCT versus CNR=1.3±0.1 for the CT. Probably this difference is due to the higher presence of noise caused by the scattered radiation. This result is consistent with the contrast‐detail curve (Fig. 8(c)) where the line shows the mean value and the colored band corresponds to the 1 σ standard deviation.

The reconstruction filter used in the CBCT affects the low‐contrast sensitivity, as can be seen in Figs. 7 and 8(a). Indeed, the higher the noise (sharp filter), the lower the low contrast sensitivity (CNR decreases and the curve shifts to higher values), and vice versa. However, the differences are not quite significant due to the uncertainty of the method (in Fig. 8(a) the standard deviation in not displayed to make the plot clearer). The results obtained are consistent with those found in the previous section, where we evaluated image noise.

Regarding the CT images, as one could expect, an increase in mAs which implies a decrease in image noise improves the CNR and shifts the contrast‐detail curve to lower values. On the other hand, reducing the size of the fan beam increases the noise and worsens the low‐contrast sensitivity, as can be seen in Figs. 7 and 8(b).

No time trend was observed.

### G. HU density curve

**Figure 9:**
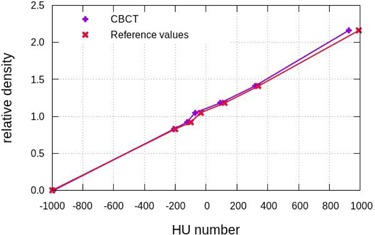
HU number versus relative density curve obtained in the CBCT (purple) with the materials present in the CTP404 Catphan 600 phantom and the reference HU numbers provided by the manufacturer (red).

The images acquired with the CBCT provide reproducible results in the measurement of HU; the standard deviation does not exceed 5 HU. In Fig. 9 we show the measured HU and the reference values provided by the manufacturer for each material. As one can see in the plot, the agreement with the reference values is good, with deviations below 35 HU, except for Teflon, where a systematic discrepancy around 60 HU is observed.

No significant dependence on the reconstruction parameters was identified. It must be noted that using a different acquisition protocol could lead to changes in the HU measurement.^(^ ^9^ ^)^ The temporal analysis has shown significant variations for some high‐density inserts: Teflon presents a variation range of 89.2 HU and Delrin 54.5 HU. For the other materials, the range of variation is below 30 HU. The data do not show any time dependence, and we think that the observed variations can be considered as statistical fluctuations.

## IV. DISCUSSION

In this paper we investigate the image quality properties of the OBI system with the CBCT protocol Pelvis. Images of the Catphan phantom were automatically analyzed by a set of macros specifically designed with the program ImageJ. The automatic analysis allows us to perform the image quality tests in a short time, which favors the routine execution of the proposed quality control. The system presents good time stability, within the analyzed three months follow‐up period. The collected data can be used as a basis for establishing the timing and tolerances in the QA.

Compared to a narrow beam CT system, the CBCT presents a less favorable geometry: larger source–detector distance and higher presence of scatter. As a consequence, one expects the appearance of a larger number of artifacts (such as cupping artifact or rings) due to the larger influence of charge trapping in the detector. In patients, one also expects streaking pattern artifacts near high‐density regions (resulting from local beam hardening effect and scattering) and movement artifact. The OBI system attempts to correct these artifacts by hardware devices or appropriate software corrections. Hence, the system incorporates a bowtie filter, antiscatter grid, nonlinear scatter correction, beam hardening correction, and ring artifact suppression algorithms. However, the resulting 3D images are not entirely free from the presence of artifacts. In fact, the images obtained from various homogeneous phantoms with different diameters have a circular band centered on isocenter with a fixed diameter of approximately 22 cm. This artifact presents a uniform intensity if the half‐fan filter is used, while it is asymmetrical in the full‐fan mode. It may be due to the vibration of the bowtie filter^(^ ^13^ ^)^ with gantry rotation. In addition, there is some nonuniformity because the cupping effect is not completely corrected. However the influence of this artifact is not very significant on the evaluation of the image quality and does not imply an important change in HU (below 50 HU).

To avoid the observer dependence in the implementation of resolution tests, we have used two physical parameters that characterize the imaging system in an objective way. Namely, the modulation transfer function for spatial resolution and the contrast‐detail ratio, calculated from statistical criteria, in the case of low‐contrast sensitivity.

The spatial resolution, calculated as the minimum size group visible in a bar pattern, reported by Kim et al.^(^ ^9^ ^)^ for a half‐fan protocol is 6 line pairs per centimeter (1p/cm), 6.2±0.4 lp/cm for Yoo et al.,^(^ ^10^ ^)^ and 4 lp/cm for Cheng et al.^(^ ^12^ ^)^ The minimum size visible group can be approximated by the 10% of the MTF curve. Therefore, according to Bissonnette et al.,^(^ ^11^ ^)^ the frequency at which the MTF reaches the 10% value is 8.4 lp/cm. With our protocol, the 10% MTF value is located approximately at 8 lp/cm.

We note that, under the conditions adopted in our study and for the same pixel size, the OBI spatial resolution is higher than that of the Aquilion, despite the smaller number of projections and the less favorable geometry. We interpret this result as a consequence of the smaller size of the detectors present in the OBI flat panel, around 194 microns, compared to the size of the detectors used by the Aquilion CT, 500 or 1000 microns.

Regarding the low‐contrast sensitivity, Cheng et al.^(^ ^12^ ^)^ found that the total number of discs visible with a nominal contrast level of 1% was 7, while targets with 0.5 and 0.3% nominal contrast were all invisible regardless of their size. Yoo et al.^(^ ^10^ ^)^ put the number of visible disc inserts in 4.2±0.4, and Kim et al.^(^ ^9^ ^)^ in 5.

According to our results, the OBI provides a low‐contrast sensitivity good enough to distinguish soft tissue. For example, the prostate usually shows a contrast level with respect to its surrounding tissue which is between 1% and 4%. The contrast‐detail curve that we have obtained indicates that the OBI is able to distinguish objects with that contrast and with a size as small as 3.3 mm. However, the correlation between the results obtained with the statistical method and those based on observers is not clear.

In summary, we conclude that the image quality is good enough to perform IGRT verifications. The implementation of ART requires a better image quality, mainly regarding the low‐contrast sensitivity. In our case, the images do seem to present an adequate visualization of the soft tissues for reliable contouring, in agreement with the clinical results published in the literature.^(^ ^3^ ^,^ ^6^ ^)^

Furthermore, in order to use the CBCT images to calculate dose distributions, it is essential that the patient contour is completely imaged. In a pelvic treatment this can only be achieved with the half‐fan mode (FOV up to 45 cm). A strict image quality control is also mandatory, especially to monitor the HU density relation, which directly affects the dose calculation. Our results suggest that this relation has a good reproducibility over time. As for the accuracy of the measured values, they are within ± 35 HU with respect to the reference values.

However, the Catphan phantom, used to measure HU, seems to present a number of drawbacks. The composition of the highest density insert, Teflon, generates a photoelectric attenuation behavior different from that of real bone,^(^ ^8^ ^)^ which makes the measured HU to be lower than real bone HU. This will affect the HU density curve. In addition, the insert size (1.2 cm diameter) seems to be too small to provide the correct HU when the amount of scattered radiation is high, as in the case of a CBCT.^(^ ^7^ ^)^

Moreover, the diameter of the Catphan phantom is 20 cm, smaller than that commonly found in clinical conditions (standard patient thickness 25–35 cm). Thus, in a real patient we expect to have more scattered radiation which, together with the presence of heterogeneities, can lead to inconsistencies in the final calculation of the dose distribution. The influence of scattered radiation on the HU density curve has been studied by Guan and Dong^(^ ^7^ ^)^ and Hatton et al.^(^ ^8^ ^)^ A variation in the scatter volume length from 5 to 26 cm can cause errors in the measurement of HU values up to 260 HU, but this seems to have an insignificant dosimetric impact.^(^ ^8^ ^)^ In addition, the calibration curve is dependent on the length of the scan — an increased z coverage will lead to more scattered radiation and, therefore, lower HU in high‐density regions. In our case, the patient scanned length will be the maximum achievable by the system (20.6 cm) in order to completely scan the treatment volume and the organs at risk. On the other hand, the radial scatter seems to present an important impact on the HU values. Differences up to 1000 HU have been found by Hatton et al.^(^ ^8^ ^)^ when varying the phantom diameter from 18 to 40 cm. Therefore, a HU density calibration with a phantom sized for each patient^(^ ^7^ ^,^ ^8^ ^)^ should be performed. However this is certainly difficult to implement in clinical practice. Another option is to perform the dose calculation with the HU density curve obtained in the narrow‐beam CT (Aquilion), which in our hospital was determined with a phantom that contained inserts with tissue‐equivalent materials. This choice has been shown to provide a good accuracy in the calculation of dose distributions on both phantoms and patients,^(^ ^2^ ^,^ ^8^ ^)^ although the degree of accuracy depends on the patient's radial diameter.^(^ ^8^ ^)^

In any case, the HU density curve obtained with the Catphan phantom is adequate to monitor the constancy of the HU density ratio in periodic quality controls.

## V. CONCLUSIONS

The OBI imaging system used as a CBCT provides volumetric images acquired with a kilovoltage cone beam. In this paper we have analyzed the image quality obtained within the Pelvis protocol, studying, in addition, its reproducibility over time. Similarly, we have analyzed the image quality provided by a simulation CT with the acquisition technique usually employed for the pelvis site in our hospital. In order to test the ability of OBI to perform IGRT and ART, we have studied some parameters that define image quality in an objective way. The analysis was automatically performed using macros implemented with the program ImageJ.

We conclude that the images obtained with the OBI system present an image quality adequate to accurately perform IGRT. However, the OBI images are noisy due to the scattered radiation present in a cone beam, and they also show some artifacts. On the one hand, we find a slight cupping effect and some ring artifacts which do not compromise the image quality. On the other hand, and of greater importance, we point out the presence of the bowtie wobble artifact. This artifact appears as a bright ring centered on the isocenter with a diameter of 22 cm. In order to carry out ART, the impact of this artifact on volume definition should be assessed. The criticality of the artifact on volume contouring will depend on the anatomical region to be imaged. The influence of the bowtie wobble artifact on dose calculation will be less important, since it involves minor changes in HU. However, before using the OBI images to perform ART, the special case of very thick patients should be considered since, in this situation, a poor choice of the HU density calibration curve could cause significant errors in dose calculation.

Finally, we stress the importance of carrying out a periodic quality control of the image used in IGRT. In addition, our experience reveals the need to calibrate the imaging system after major interventions, and then check the quality of the obtained images. This becomes more important if we intend to use the OBI to perform ART.
